# It is time to explore the impact of length of gestation and fetal health on the human lifespan

**DOI:** 10.1111/acel.14157

**Published:** 2024-04-01

**Authors:** Zhuo Yu, Yushan Dong, Yuhan Chen, Lotfi Aleya, Yinhuan Zhao, Lan Yao, Weikuan Gu

**Affiliations:** ^1^ Heilongjiang Academy of Traditional Chinese Medicine Harbin China; ^2^ Graduate School of Heilongjiang University of Chinese Medicine Harbin Heilongjiang China; ^3^ Guang'anmen Hospital, China Academy of Chinese Medical Sciences Beijing China; ^4^ Chrono‐Environnement Laboratory, UMR CNRS 6249 Bourgogne Franche‐Comté University Besançon Cedex France; ^5^ Department of Rheumatism, Shanghai Traditional Chinese Medicine Integrated Hospital Shanghai University of Traditional Chinese Medicine Shanghai China; ^6^ College of Health Management, Harbin Medical University Harbin Heilongjiang China; ^7^ Department of Orthopedic Surgery and BME‐Campbell Clinic University of Tennessee Health Science Centre Memphis Tennessee USA; ^8^ Research Lt. Col. Luke Weathers Jr. VA Medical Center Memphis Tennessee USA; ^9^ Department of Pharmaceutical Sciences University of Tennessee Health Science Center Memphis Tennessee USA

**Keywords:** body growth, body size, gestation, lifespan, longevity

## Abstract

A recently proposed principal law of lifespan (PLOSP) proposes to extend the whole human lifespan by elongating different life stages. As the preborn stage of a human being, gestation is the foundation for the healthy development of the human body. The antagonistic pleiotropy (AP) theory of aging states that there is a trade‐off between early life fitness and late‐life mortality. The question is whether slower development during the gestation period would be associated with a longer lifespan. Among all living creatures, the length of the gestation period is highly positively correlated to the length of the lifespan, although such a correlation is thought to be influenced by the body sizes of different species. While examining the relationship between lifespan length and body size within the same species, dogs exhibit a negative correlation between lifespans and body sizes, while there is no such correlation among domestic cats. For humans, most adverse gestational environments shorten the period of gestation, and their impacts are long‐term. While many issues remain unsolved, various developmental features have been linked to the conditions during the gestation period. Given that the length of human pregnancies can vary randomly by as long as 5 weeks, it is worth investigating whether a slow steady healthy gestation over a longer period will be related to a longer and healthier lifespan. This article discusses the potential benefits, negative impacts, and challenges of the relative elongation of the gestation period.

## Graphical abstract

Between the birth at 37 and 42 weeks of gestation, a baby may be different in the health and growing process. The effect factors and the impact of length of gestation on late life development and aging should be explored in our future research.

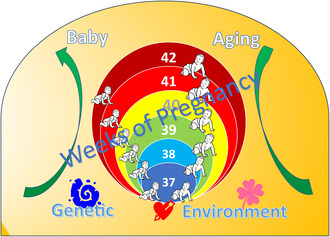

**Abbreviations**
AP: antagonistic pleiotropy
BMI: body mass index
GWG: gestational weight gain
IGFs: insulin‐like growth factors
PLOSP: proposed principal law of lifespan
T3: triiodothyronine
T4: thyroxine

## 1 INTRODUCTION

### 1.1 Why gestation and biological status affect the whole lifespan

While a tremendous volume of research has explored the gestation and reproductivity of humans, the study on the relationship between gestation and the entire lifespan has been left blank. It has never been asked whether an elongation of the period of gestation and slowing down the development of a preterm fetus may lead to a longer and healthier lifespan. Recently, a new research based on the principal law of lifespan (PLOSP) to extend human lifespan has been proposed (Gu, 2022a). While the hypothesis remains to be evaluated, one valued argument from the PLOSP theory is that more effort should be put into examining the effect of elongating different life stages.

So far, most of the biomedical research on the extension of the lifespan has focused on prolonging the aging stage, that is, improving the health of the elderly. However, this approach is reaching its plateau, given that the annual growth rate of the lifespan in the United States has decreased from approximately 0.3% 50 years ago to approximately 0.05% in recent years (Gu, 2022a). Compared to earlier life stages, prolonging the aging stage is considered a less favorable approach of extending the lifespan, in the light of the deterioration of the aging body's functions at this stage of life (Gu, 2022b). Because life is a continuing process with the early stage laying the foundation for the next, the health status of the earlier life stages ultimately benefits the fitness during the aging stage. For example, the health status in gestation is known to affect the body growth and development of adults (Deter et al., 1999; Gicquel & Bouc, 2006; Weiss, 2000; Mitteldorf, 2019; Solé‐Navais et al., 2023; Ricklefs, 2010). We argue that emphasizing the research on the length of the gestation period and its impact on the lifespan diverts some resources used for research on other life stages, including the aging stage.

### 1.2 Biological mechanisms involved in the regulation of fetal development

**FIGURE 1:**
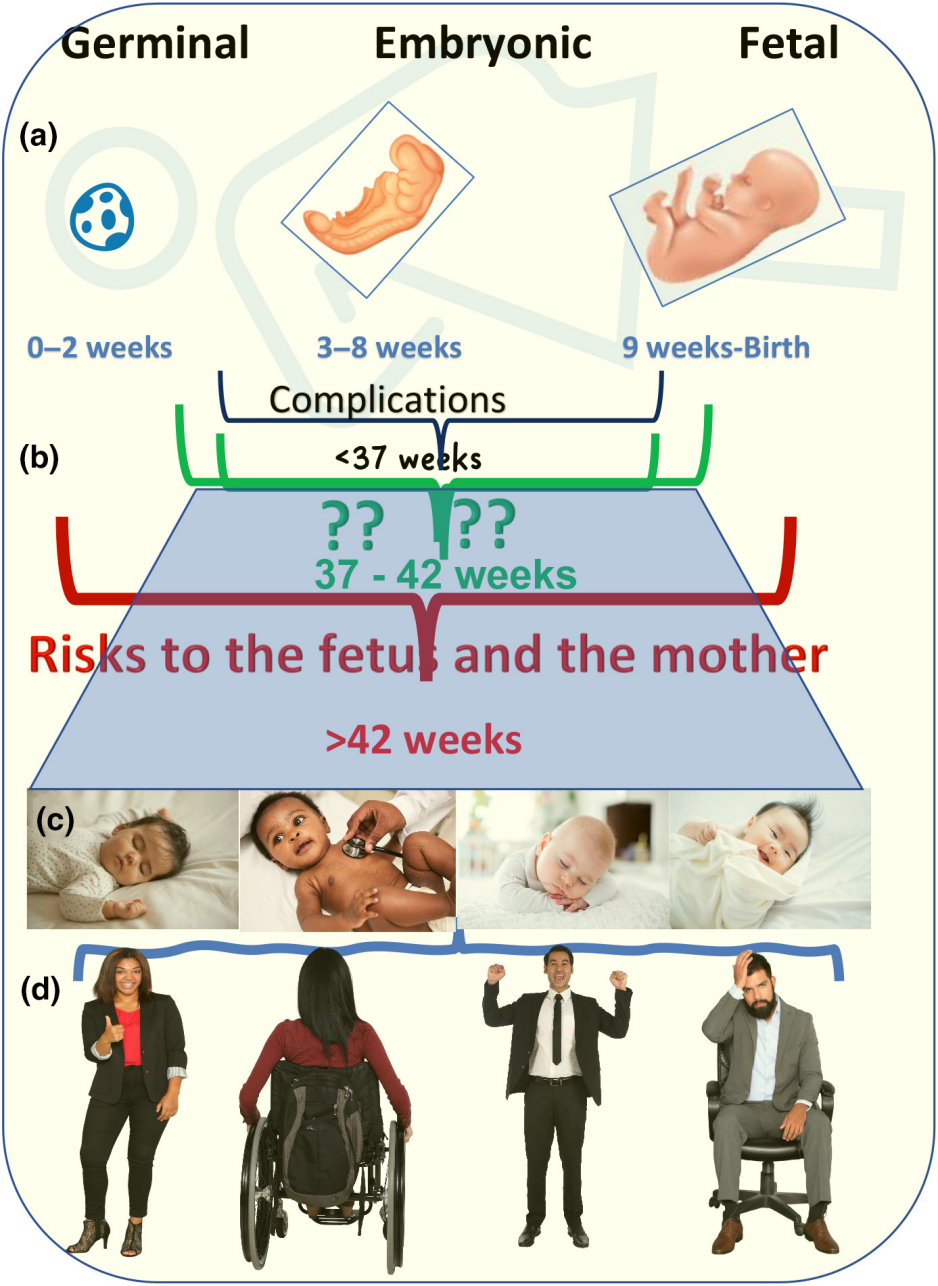
Human fetal development and length of gestation period. (a) Three stages of the gestation period. (b) Current definitions of pre‐term birth (<37 weeks), term birth (37–42 weeks), and post‐term birth (>42 weeks) are based on the length of gestation. (c) Potential variation of neonate body weight and health status among neonates born within the normal range for gestation period. (d) Potential impact of gestation length on body growth and lifespan among the adult population.

The first stage of human life, fetal development, is divided into three stages: the germinal, embryonic, and fetal stages (Figure 1a). The germinal stage starts at the time of conception when the sperm and egg combine to form a zygote until implantation is complete. This is followed by the embryonic stage, during which the organ systems will begin to form and develop. The stage from then until birth is the fetal stage. The length of gestation among human populations varies considerably, mainly due to the difference in growth rates during the embryonic and fetal stages of development (Deter et al., 1999). Hormones such as insulin‐like growth factors (IGFs), cortisol, and thyroxine play a key role in regulating fetal growth and development, acting in concert to ensure that the fetal growth rate matches the nutrient supply and that prepartum maturation occurs in preparation for extrauterine life (Gicquel & Bouc, 2006). Environmental factors, such as the mother's health status, mood, and living conditions affect the levels of hormones and the development of the fetus. Thus, the length of gestation is regulated by the complicated interactions between genetic and environmental factors. The question remains within this variation in the gestational period as to how its length is related to the lifespan of that species.

### 1.3 Lesson from the study of the parturition cascade

Variations in gestational length between species may be due to different parturition mechanisms in each species. There are considerable studies on the parturition cascade (Weiss, 2000), which triggers the induction of labor at term (37–42 weeks in humans). The balance between the effects of estrogen and progesterone is critical to the maintenance of pregnancy and the onset of labor. The timely onset of labor and birth determines the perinatal outcome. Both preterm birth (delivery before 37 weeks of pregnancy) and post‐term pregnancy (failure to deliver before 42 weeks of pregnancy) are associated with an increased risk of adverse pregnancy events. The normal period of onset of labor occurs between 37 and 42 weeks (Figure 1b). An essential question is whether the length of gestation has any effect on the speed of body growth, the timing of sexual maturity, and eventually the length of the lifespan for individuals in the human population (Figure 1c,d).

## 2 HYPOTHESIS

Based on the PLOSP, the human lifespan could be significantly extended and healthier if the different life stages were to be elongated with specific strategies (Gu, 2022a). The antagonistic pleiotropy (AP) theory of aging posits that there is a trade‐off between early life fitness and late‐life mortality (Austad & Hoffman, 2000). Recent studies suggest that maternal alleles that increase the gestational duration have negative fetal effects, leading to low birth weight in the human population (Ricklefs, 2010; Solé‐Navais et al., 2023).In humans, the relationship between the length of gestation and lifespan has not been thoroughly studied. We hypothesize that within the normal range of variation, the length and growth speed of healthy gestation positively correlate with the length and quality of the human lifespan. The proposed elongation of the gestation period will allow the steady and healthy slow growth of the developing fetus but would not be appropriate in unhealthy situations or disease conditions. Instead, the hypothesis refers to the lifespan of healthy individuals born at different lengths of gestation, which has not been studied previously.

## 3 DISCUSSION

### 3.1 Evidence from animal studies

One study that compared the life‐history data of ~800 mammalian species found positive correlations between maximum lifespan and gestation period and, at the same time, between body size and lifespan (Fushan et al., 2015). There are considerable available data on the gestation period and lifespan of mammals (Ricklefs, 2010; Beef2Live; Krog et al., 2018; Wu et al., 2021; FutureLearn, 2023; Vallet et al., 2013). A few studies showed a potential positive relationship between them. For example, Wu et al. (2021) describe a multifactorial model in which partial correlations were created for 89 mammalian species between multiple factors such as gestation length, litter size, time to independence, age of female sexual maturity, weaning period, and food availability. In this model, gestation length and lifespan have the strongest correlation (Wu et al., 2021). Alternatively, Ricklefs (2010) showed a negative correlation between the rate of aging and the length of the gestation period.

**FIGURE 2:**
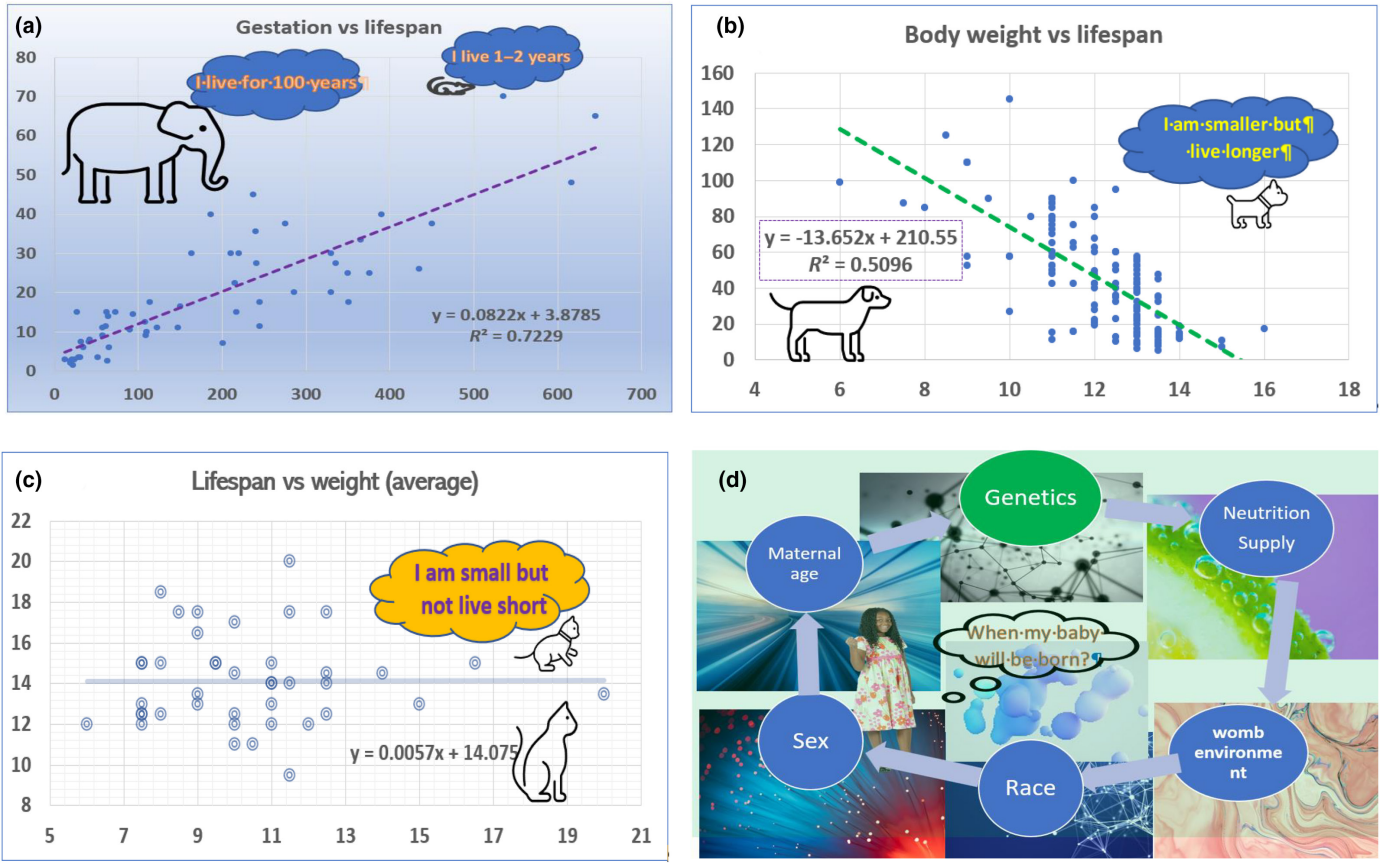
Gestation period, body weight, and lifespan among mammals. (a) There is a positive correlation between the length of gestation periods and lifespan among mammals. While mammals with longer gestation periods generally have longer lifespans, most of them also have larger body sizes relative to mammals with short lifespans. The y‐axis indicates the lifespan in years, while the x‐axis indicates the length of the gestation period in days. (b) There is a negative correlation between lifespan length and body size among different breeds of domestic dogs. (c) The lack of correlation between the lifespan lengths and body sizes in domestic cats of different breeds. The x‐axis indicates the lifespan in years, while the y‐axis indicates the body weight in pounds. (d) Environmental conditions affect the length of gestation period.

We collected data on gestation length and lifespan for 59 mammalian species and analyzed the regression coefficient (*R*) among them (Table S1) using the CORREL formula in Excel. The result showed a strong positive correlation between the average length of the gestation period and the average length of lifespan among these species (Figure 2a). At first glance, when considering the list of animals we assembled, one would claim that the ones with longer lifespans also have larger body sizes. Similarly, Phillippe and Phillippe (2017) suggested that there was a positive relationship between the length of the gestation period and the length of the lifespan in mammals.

The data for different breeds of domestic dogs and cats provides an excellent opportunity to perform analysis. Data for 133 breeds of domestic dogs with great variation in body weight (ranging from 6 to 110 pounds) and lifespan (ranging from 6 to 16 years) were collected from open‐use databases (American Kennel Club, 2023; PetCareRx, 2023a), as shown in Table S2. Surprisingly, we found a negative correlation between body sizes and lifespans among these dogs, with a *r* value of −0.71 (*R* ^2^ = 0.51), as shown in Figure 2b. There is also body weight and lifespan data available for domestic cat breeds from a variety of sources (Cat and Friends, 2023; PetCareRx, 2023b). Compared to dogs, cats exhibit a lower variation in body weight and lifespan (Table S3). By comparing the average body weight with the average lifespan of 41 cat breeds, we did not find any significant correlation between body weight and lifespan (Figure 2c). Although more evidence may be needed, the data from dogs and cats strongly suggest that the gestation length within the same species does not correlate with body weight. The variation in the days of gestation within the same species is known in a dozen animal species (Declercq et al., 2023; Okkens et al., 2001; Wu et al., 2021).

### 3.2 Indirect evidence from human studies

In Sweden, Quaranta et al investigated the size at birth and the longevity of the population using archival data on birth characteristics and gestational age (but not the length of gestation period) from 995 live singleton full‐term births at the Halmstad Hospital, Halland, and compared them to 3364 births from three hospitals in nearby Scania (Quaranta et al., 2022). The authors concluded that the longer mean life expectancy in Halland compared to the rest of Sweden could have been associated with beneficial early life factors that influenced birth size.

Other studies whose results may have such a relationship did not approach this question directly. In a review of the metabolic imprinting of choline by its availability during gestation, Meck and Williams (2003) reported that the perinatal period is a critical time for the cholinergic organization of brain function and that supplementation with choline during the gestation period appears to prevent age‐related memory and attentional decline.

In 2023, the life expectancy in the United States was 77.3 years, while in the Netherlands it was 81.1 years and 80.6 years in the UK (Life Expectancy of the World Population, 2023). In a study comparing gestational age and birth timing in these three high‐income countries, Declercq et al. (2023) reported that in 2020, only 23% of US hospital births occurred at 40 or more weeks gestation, compared to 44% of hospital births in the Netherlands and 40% of hospital births in the UK. These data point to a potential correlation between the length of the gestation period and the life expectancy (length of lifespan). The length of the human gestation period can be varied as many as 5–6 weeks (Jukic et al., 2013). Some studies on gestation focus on early body growth and neurological development (Espel et al., 2014; Meck & Williams, 2003). Coincidentally, other studies have suggested that some neuromotor reflexes are more mature at delivery if a fetus has been gestated longer (Coe & Lubach, 2021; Kelleher et al., 2017).

### 3.3 Factors that influence the length of gestation

If the unfavorable conditions that can lead to premature or postmaturity birth are removed, neonates born at various times within the normal gestation period can exhibit considerable changes (Figure 2d). The normal length of gestation (37–42 weeks) can vary by a time difference of more than 10%. The speed of growth of a fetus in the mother's womb can be influenced by an extremely complicated mixture of multiple genetic and environmental factors, including maternal nutritional status and physiologic hyperinsulinemia (Coe & Lubach, 2021; Price et al., 1999; Quaranta et al., 2022; Riopelle & Hale, 1975; Susa et al., 1984).

Studies of human populations and animal models indicate that many adverse pregnancy outcomes, including congenital anomalies, increased risk for miscarriage or pre‐term delivery, intrauterine growth restriction, and stillbirth, are accompanied by shorter rather than longer periods of gestation (Chersich et al., 2020; Ozturk et al., 2016; Yuasa et al., 2016). Some of the associated environmental factors can also lead to physical or emotional conditions in the developing fetus, the neonate, or even the adult they become (Fleming et al., 2018; Marshall et al., 2022; Mbiydzenyuy et al., 2022; Moisiadis & Matthews, 2014; Muglia et al., 2022). Accordingly, these authors recommended the new guidance on parental preparation for pregnancy that begins before conception to protect the health of their offspring. Bergeron et al. (2023) found that shorter length of gestation in the preterm and early term intervals led to emotional distress in the offspring. Thus, while adverse gestational environments can have a lifelong impact on the offspring (Hopper et al., 2008), most such cases lead to pre‐term or early term births (Bergeron et al., 2023; Chersich et al., 2020; Ozturk et al., 2016; Yuasa et al., 2016). This may be interpreted as suggestive evidence that supports our hypothesis.

Growth rates of different organ systems are different within a single species and among different species. In humans, most organs are completely formed by approximately 12 weeks of pregnancy, while the brain and spinal cord continue to form and develop throughout pregnancy (Kalusa et al., 2021; Molnár & Clowry, 2012). The speed of brain development differs for other species. Rodents exhibit accelerated postnatal development of the brain (Dehorter et al., 2012). Several studies have explored the effect of environmental factors and sex differences on development (Jukic et al., 2013; McCarthy, 2016; Meck & Williams, 2003; Okkens et al., 2001; Riopelle & Hale, 1975) and reports in the literature indicate the effect of a healthy gestation period on the health and learning ability of the neonate (Black et al., 2022; Klejnstrup et al., 2018; Lampl et al., 2009; Nolan et al., 2017; Norris et al., 2022; Victora et al., 2022).

As hormones regulate fetal growth and development (Gicquel & Bouc, 2006), one might speculate whether manipulation of hormone levels will increase the length of the gestation period and speed of fetal growth. For instance, insulin‐like growth factors (IGFs) cortisol and thyroxine (T4) and triiodothyronine (T3) control placental resource allocation to fetal growth, differentiation and maturation, and metabolism, response to adverse gestational environments and length of gestation (Forhead et al., 2006; Forhead & Fowden, 2014; Fowden & Forhead, 2022; Muhammad et al., 2020; Owens, 1991; Sferruzzi‐Perri et al., 2017; Yu et al., 2022).

Genomic elements and epigenetic factors have been suggested to play roles in the length of the gestation period, including the polymorphisms in regulatory genes (Jinno et al., 2020; Moslehi et al., 2010), circulating microRNAs (Martinez‐Fierro & Garza‐Veloz, 2021), the length of chromosomal telomeres (Goumy et al., 2022), and other developmental features (Gafner et al., 2023; Workalemahu et al., 2018).

### 3.4 Future work and challenges

The proposed hypothesis is based on an important assumption, that the length of gestation depends on the maturity of the fetus such that all neonates are born in similar development stages, despite the variation of 5–6 weeks in the length of the gestation period. The critical issue is that there is currently no definite answer as to what determines the time for a developing fetus to be born.

Maternal body mass index (BMI) and gestational weight gain (GWG) affect the outcomes of pregnancy (Barisic et al., 2017), with GWG during pregnancy increasing the size of the developing fetus as well as the risk of stillbirth (Woolner & Bhattacharya, 2015). Unfortunately, overweight or obese populations have become the new “normal” worldwide (Lindsay et al., 2022). Thus, in the case of excessive maternal weight while pregnant, elongating the period of gestation might increase the birth weight of the neonate and the risk of stillbirth. While advanced maternal age during pregnancy is considered high risk and can negatively impact both mother and fetus (Correa‐de‐Araujo & Yoon, 2021), young maternal age is also associated with poorer birth outcomes (Di Gravio et al., 2019). How to manipulate the length of the gestation period and avoid the adverse effects of unfavorable birth outcomes and health challenges in later life should be addressed in future studies.

Extending the length of the gestation period beyond its normal range of variation will bring tremendous obstacles, including ethical issues. One ethical question is that some people may not want to live such a long time while others may want to do so. The extension of the lifespan by removing the limits of different life stages provides the opportunity for people who would like to live longer. Overall, the concept of manipulating life stages would provide the opportunity for people to choose between living longer or the longer early life stage.

There are also safety issues that might require consideration before prolonging the gestation period. First, there may be concerns about whether manipulating the length of the gestation period would affect the health status of the mother. Some research stated that the manipulation of gestation length is too risky since pregnancy is such a unique period for each species that it can be affected by many factors (Khan & Leventhal, 2022; Uttara et al., 2016). Although our hypothesis is for the manipulation of gestation length for women who are healthy at the onset of pregnancy, the manifestation of disease or illness later in the pregnancy cannot be ruled out.

It is unknown whether manipulation of the growing uterine environmental conditions in which the fetus develops will cause the fetus to grow at a slower speed or whether it will bring about a longer lifespan. Assuming that the speed of development of a human being will continue its slow pace after birth, at least until the adolescent life stage, then such extension of gestation might result in the lengthening of the human lifespan. Certainly, speeding up fetal growth leads to preterm birth (Lampl et al., 2009), while a slow growth velocity of the fetal femur is associated with an increased risk of spontaneous preterm birth (Sferruzzi‐Perri et al., 2017). Thus, there are many challenges to overcome in slowing down the fetal growth speed while maintaining the health status of neonates as they grow to adulthood.

Working on the other life stages will most likely achieve the goal of increasing the lifespan of humans (Gu, 2022a), yet many issues remain to be solved (Chen et al., 2022; Yao et al., 2022). For example, while estrogen is responsible for the development and regulation of the female reproductive system, manipulating levels of estrogen and other relevant growth factors is a complex issue that should be based on individual characterization of the life stages (Kajantie, 2008; Wang et al., 2022; Yu et al., 2022). Similarly, the decrease in the level of testosterone among aging men is slow and steady but the relative level varies greatly among male populations (Wang et al., 2022). Therefore, personalized manipulation of testosterone levels at different life stages in men will also be a complicated task.

Parturition also plays a critical role in pregnancy and is thus related to the length of the gestation period. If all neonates were born after gestation periods of the same length, would a neonate with a heavier body weight have a longer lifespan? Or would a neonate with a longer gestation period and a heavier body weight have a longer lifespan than a neonate born with a shorter gestation period and less body weight? Many questions remain.

## 4 CONCLUSIONS

Many questions remain before we can entertain such a possibility. We hope that this article serves as the beginning of a new phase of study on the relationship between gestation and life expectancy. We recommend that such studies start with testing the hypothetical association between the length of the gestation period and that of the human lifespan. We have presented mostly indirect evidence for the potential positive association between the length of the gestation period and the length of lifespan. A systematic, direct comparison between the length of the gestation period and the length of the lifespan will be essential to test or confirm our hypothesis. Such a relationship can be examined from the population of dogs and cats with available data because: (1) the length of the lifespan varies among different breeds and animals of the same breed; (2) the length of the gestation period varies among different breeds and animals of the same breed; and (3) the status of dogs and cats as favorite human pets means that there are large populations and reams of data available for these studies.

In humans, such a study might be performed using data from different populations within different environments but would face great challenges. Theoretically, determining the associations between the length of gestation and the length of the lifespan will require a large dataset of the population, including the lengths of gestation periods and the age at death. The data should be from the same population and with the same environmental conditions. If such information can be obtained from existing populations of genome‐wide association studies or reference populations, the hypothesis can be tested by examining the correlation between the length of gestation and the age at death. It might be easier to obtain gestation period data from younger populations, but it would take a long time to follow up with the participants. Beyond supporting or refuting our hypothesis, these studies should include the safety of the mother and the fetus. The data generated from these studies should be accompanied by the development of public policies and regulations under which the gestation length could be manipulated. The validation and application of methodologies to manipulate the length of gestation period should be studied first in animal models before being applied to human beings.

Given the consideration of the concept of PLOSP, a total lifespan is comprised of different life stages (1), each of which would provide an opportunity to elongate the whole lifespan. While we recognize the role of the body growth and reproduction stages, it remains important to highlight the life stage that comprises the gestation period. The impact of the speed of fetal growth on the late life stages may be far beyond our knowledge. As we continue to explore the impact of different life stages on the total lifespan, its extension will enter a new phase that occurs more rapidly than its current speed.

## AUTHOR CONTRIBUTIONS

WG developed the concept. ZY, YD, LY, and WG participated in data analysis and manuscript drafting. YC, LA, LY, and YZ interpreted the data and edited the manuscript.

## CONFLICT OF INTEREST STATEMENT

The authors declare no competing financial interests.

## Supporting information


Table S1.

## Data Availability

All data have been presented in the manuscript and supplemental materials and are available for public.
