# Supplementary material for: It is time to explore the impact of length of gestation and fetal health on the human lifespan
Source: Aging Cell. 2024 Apr 1;23(4):e14157. doi: 10.1111/acel.14157 (PMC11019132; doi:10.1111/acel.14157)
Supplement: Supplementary file 1 — Table S1. [file ACEL-23-e14157-s001.docx]

Supplementary Table 1. Relationship between length of gestation period and lifespan in mammals.

| Mammal | Gestation period (days) | Average Gestation period (days) | Lifespan | Average lifespan (years) |
| --- | --- | --- | --- | --- |
| Alpaca | 335-366 | 350.5 | 15-20 | 17.5 |
| Baboon | 187 | 187 | 35-45 | 40 |
| Bear, black | 220 | 220 | 30 | 30 |
| Bear, grizzly | 215 | 215 | 20-25 | 22.5 |
| Bear, polar | 241 | 241 | 25-30 | 27.5 |
| Beaver | 122 | 122 | 10-12 | 11 |
| Bison, American | 217 | 217 | 10-20 | 15 |
| Camel, Bactrian | 360-420 | 390 | 40 | 40 |
| Cat, domestic | 58–67 | 64 | 12-18 | 15 |
| Chimpanzee | 230–250 | 240 | 32-39 | 35.5 |
| Chinchilla | 105–115 | 110 | 10 | 10 |
| Chipmunk | 31 | 31 | 2-5 | 3.5 |
| Cow | 279–292 | 285.5 | 20 | 20 |
| Deer, white-tailed | 201 | 201 | 6-8 | 7 |
| Dog, domestic | 58–65 | 61 | 10-13 | 11.5 |
| Donkey | 365 | 365 | 27-40 | 33.5 |
| Elephant, Asian | 617 | 617 | 48 | 48 |
| Elephant, African | 645 | 645 | 60-70 | 65 |
| Elk, Wapiti | 240–250 | 245 | 10-13 | 11.5 |
| European mink | 38-76 | 57 | 8-10 | 9 |
| Ferret, domestic | 41-42 | 41.5 | 5-10 | 7.5 |
| Fox, red | 52 | 52 | 3-4 | 3.5 |
| Gerbil | 22-26 | 24 | 2-4 | 3 |
| Giraffe | 420–450 | 435 | 26 | 26 |
| Goat, domestic | 145–155 | 150 | 15-18 | 16.5 |
| Gorilla | 255-260 | 275 | 35-40 | 37.5 |
| Guinea pig | 56-74 | 65 | 4-8 | 6 |
| Hamster | 16-23 | 19.5 | 2-3 | 2.5 |
| Hippopotamus | 225–250 | 237.5 | 40-50 | 45 |
| Horse | 330–342 | 336 | 25-30 | 27.5 |
| Human | 266 | 266 |  |  |
| Kangaroo | 42 | 42 | 8 | 8 |
| Leopard | 92–95 | 93.5 | 12-17 | 14.5 |
| Lion | 108 | 108 | 10-15 | 12.5 |
| Llama | 330 | 330 | 20 | 20 |
| Mink | 40–75 | 57.5 | 10-12 | 11 |
| Monkey, rhesus | 164 | 164 | 30 | 30 |
| Moose | 240–250 | 245 | 15-20 | 17.5 |
| Mouse | 19 | 19 | 2 | 2 |
| Mouse, meadow | 21 | 21 | 3 | 3 |
| Muskrat | 28–30 | 29 | 3-4 | 3.5 |
| Opossum, Virginia | 12–13 | 12.5 | 2-4 | 3 |
| Otter | 60-86 | 73 | 10-20 | 15 |
| Pig, domestic | 112–115 | 113.5 | 15-20 | 17.5 |
| Porcupine | 210 | 210 | 30 | 30 |
| Puma | 90 | 90 | 8-13 | 10.5 |
| Rabbit, domestic | 28–35 | 31.5 | 5-10 | 7.5 |
| Raccoon | 63 | 63 | 2-3 | 2.5 |
| Rat | 21-23 | 22 | 1-2 | 1.5 |
| Rhinoceros, black | 450 | 450 | 35-50 | 37.5 |
| Seal | 330 | 330 | 30 | 30 |
| Sea lion, California | 350 | 350 | 20-30 | 25 |
| Sheep, domestic | 144–151 | 147.5 | 10-12 | 11 |
| Squirrel, gray | 30–40 | 35 | 6 | 6 |
| Tiger | 105–113 | 109 | 8-10 | 9 |
| Whale, sperm | 480–590 | 535 | 70 | 70 |
| Wolf | 60–68 | 64 | 14 | 14 |
| Wombat | 26–28 | 27 | 15 | 15 |
| Zebra, Grant's | 361-390 | 375.5 | 20-30 | 25 |
| R = 0.850216 | | | | |

Supplementary Table 2. Relative lifespan and body weight in domestic dog breeds.

| Domestic Dog Breed | Lifespan (Years) | Average Lifespan | Average Body Weight (Pounds) | |
| --- | --- | --- | --- | --- |
|  |  |  | Male | Female |
| Affenpinscher | 12-14 | 13 | 8.5 | 8.5 |
| Afghan Hound | 12-14 | 13 | 55 | 55 |
| Airedale Terriers | 10-13 | 11.5 | 60 | 60 |
| Akitas | 10-13 | 11.5 | 120 | 85 |
| Alaskan Malamutes | 10-13 | 11.5 | 85 | 75 |
| African Boerboels | 9-11 | 10 | 175 | 175 |
| American Eskimo Dog | 12-14 | 13 | 30 | 30 |
| American Foxhound | 10-13 | 11.5 | 67.5 | 62.5 |
| American Staffordshire Terrier | 12-14 | 13 | 62.5 | 47.5 |
| American Water Spaniel | 10-12 | 11 | 37.5 | 32.5 |
| Anatolian Shepherd Dog | 10-13 | 11.5 | 130 | 100 |
| Australian Cattle Dog | 10-13 | 11.5 | 42.5 | 42.5 |
| Australian Shepherd | 12-15 | 13.5 | 57.5 | 47.5 |
| Australian Terrier | 12-14 | 13 | 17.5 | 17.5 |
| Basenji | 12-14 | 13 | 24 | 22 |
| Basset Hound | 11-14 | 12.5 | 52.5 | 52.5 |
| Beagle | 12-14 | 13 | 25 | 25 |
| Bearded Collie | 12-14 | 13 | 50 | 50 |
| Beauceron | 10-12 | 11 | 90 | 90 |
| Bedlington Terrier | 12-14 | 13 | 20 | 20 |
| Belgian Malinois | 10-12 | 11 | 70 | 50 |
| Belgian Shepherd Dog | 10-12 | 11 | 65 | 52.5 |
| Belgian Tervuren | 10-12 | 11 | 65 | 52.5 |
| Bernese Mountain Dog | 6-9 | 7.5 | 117.5 | 82.5 |
| Bichon Frise | 12-15 | 13.5 | 15 | 15 |
| Black and Tan Coonhound | 10-12 | 11 | 87.5 | 87.5 |
| Black Russian Terrier | 10-11 | 10.5 | 105 | 105 |
| Bloodhound | 10-12 | 11 | 100 | 90 |
| Border Collie | 10-14 | 12 | 42.5 | 42.5 |
| Border Terrier | 12-15 | 13.5 | 14.25 | 12.75 |
| Borzoi | 10-12 | 11 | 90 | 72.5 |
| Boston Terrier | 14 | 14 | 18.5 | 18.5 |
| Bouvier des Flandres | 10-12 | 11 | 90 | 90 |
| Boxer | 8-10 | 9 | 72.5 | 57.5 |
| Briard | 10-12 | 11 | 77.5 | 77.5 |
| Brittany | 12-13 | 12.5 | 35 | 35 |
| Brussels Griffon | 12-15 | 13.5 | 9 | 9 |
| Bull Terrier | 11-14 | 12.5 | 60 | 60 |
| Bullmastiff | 8-10 | 19 | 120 | 110 |
| Cairn Terrier | 12-14 | 13 | 14 | 13 |
| Canaan Dog | 12-13 | 12.5 | 50 | 40 |
| Cane Corso | 11 | 11 | Prop. to ht | Prop. to ht |
| Cardigan Welsh Corgi | 12-14 | 13 | 34 | 29.5 |
| Cavalier King Charles Spaniel | 9-14 | 11.5 | 15.5 | 15.5 |
| Cesky Terrier | 14 | 14 | 19 | 19 |
| Chesapeake Bay Retriever | 10-13 | 11.5 | 72.5 | 62.5 |
| Chihuahua | 14-18 | 16 | < 6 | < 6 |
| Chinese Crested | 13-15 | 14 | 10 | 10 |
| Chinese Shar-Pei | 8-10 | 9 | 52.5 | 52.5 |
| Chow Chow | 8-12 | 10 | 57.5 | 57.5 |
| Clumber Spaniel | 10-12 | 11 | 77.5 | 62.5 |
| Cocker Spaniel | 12-15 | 13.5 | 27.5 | 22.5 |
| Collie | 8-12 | 10 | 67.5 | 57.5 |
| Coton De Tulears | 15 | 15 | 12 | 10.5 |
| Curly-Coated Retriever | 8-12 | 10 | 77.5 | 77.5 |
| Dachshund | 12-14 | 13 | 24 | 24 |
| Dalmatian | 12-14 | 13 | 57.5 | 57.5 |
| Dandie Dinmont Terrier | 11-13 | 12 | 21 | 21 |
| Doberman Pinscher | 10-12 | 11 | 87.5 | 75 |
| Dogue de Bordeaux | 5-7 | 6 | 110 + | 99 + |
| English Cocker Spaniels | 12-14 | 13 | 31 | 29 |
| English Foxhound | 10-13 | 11.5 | 67.5 | 67.5 |
| English Setter | 10-12 | 11 | 72.5 | 50 |
| English Springer Spaniel | 10-14 | 12 | 50 | 40 |
| English Toy Spaniel | 10-12 | 11 | 11 | 11 |
| Field Spaniel | 12-14 | 13 | 42.5 | 42.5 |
| Finnish Spitz | 12-14 | 13 | 29 | 24 |
| Flat-Coated Retriever | 10-13 | 11.5 | 65 | 65 |
| Fox Terrier (Smooth) | 10-13 | 11.5 | 18 | 16 |
| Fox Terrier (Wire) | 10-13 | 11.5 | 18 | 16 |
| French Bulldog | 9-11 | 10 | > 28 | > 28 |
| German Pinscher | 12-15 | 13.5 | 35 | 35 |
| German Shepherd | 10-12 | 11 | 62.5 | 60 |
| Giant Schnauzer | 10-12 | 11 | 72.5 | 65 |
| Glen of Imaal Terrier | 10-14 | 12 | 36 | 36 |
| Golden Retriever | 10-13 | 11.5 | 70 | 60 |
| Gordon Setter | 10-12 | 11 | 67.5 | 57.5 |
| Great Dane | 7-10 | 8.5 | 157.5 | 125 |
| Great Pyrenees | 10-12 | 11 | 100 + | 85 + |
| Greater Swiss Mountain Dog | 10-12 | 11 | 127.5 | 97.5 |
| Greyhound | 10-13 | 11.5 | 67.5 | 62.5 |
| Harrier | 12-14 | 13 | 52.5 | 52.5 |
| Havanese | 12-14 | 13 | 10 | 10 |
| Ibizan Hound | 12-14 | 13 | 50 | 45 |
| Irish Setter | 12-14 | 13 | 70 | 60 |
| Irish Terrier | 12-15 | 13.5 | 27 | 25 |
| Irish Water Spaniel | 10-13 | 11.5 | 61.5 | 51.5 |
| Irish Wolfhound | 5-7 | 6 | 120 | 105 |
| Italian Greyhound | 12-15 | 13.5 | 10.5 | 10.5 |
| Jack Russell Terrier | 13 | 13 | 12 | 12 |
| Japanese Chin | 12-14 | 13 | 9 | 9 |
| Keeshond | 12-14 | 13 | 40 | 40 |
| Kerry Blue Terrier | 12-15 | 13.5 | 36.5 | Prop. less |
| Komondor | 10-12 | 11 | 100 + | 80 + |
| Kuvasz | 9-12 | 10.5 | 107.5 | 80 |
| Labrador Retriever | 10-12 | 11 | 72.5 | 62.5 |
| Lakeland Terrier | 12-16 | 14 | 17 | Sl. smaller |
| Lhasa Apso | 12-14 | 13 | 15 | 15 |
| Löwchen | 13-15 | 14 | 15 | 15 |
| Maltese | 12-14 | 13 | < 7 | < 7 |
| Manchester Terrier | 15-16 | 15.5 | 17 | 17 |
| Mastiff | 9-11 | 10 | 195 | 145 |
| Miniature Bull Terrier | 11-14 | 12.5 | 24 | 24 |
| Miniature Pinscher | 12-14 | 13 | 9 | 9 |
| Miniature Schnauzer | 12-14 | 13 | 15.5 | 15.5 |
| Neapolitan Mastiff | 8-10 | 9 | 150 | 110 |
| Newfoundland | 8-10 | 9 | 140 | 110 |
| Norfolk Terrier | 13-15 | 14 | 11.5 | 11.5 |
| Norwegian Buhunds | 11-13 | 12 | 35.5 | 30.5 |
| Norwegian Elkhound | 10-12 | 11 | 55 | 48 |
| Norwich Terrier | 13-15 | 14 | 12 | 12 |
| Nova Scotia Duck Tolling Retriever | 11-13 | 12 | 42.5 | 42.5 |
| Old English Sheepdog | 10-12 | 11 | 80 | 80 |
| Otterhound | 10-13 | 11.5 | 115 | 80 |
| Papillon | 12-15 | 13.5 | 7.5 | 7.5 |
| Parson Russell Terrier | 13-15 | 14 | 15 | 15 |
| Pekingese | 12-15 | 13.5 | < 14 | < 14 |
| Pembroke Welsh Corgi | 11-13 | 12 | < 30 | < 28 |
| Petit Basset Griffon Vendeen | 11-14 | 12.5 | 32.5 | 32.5 |
| Pharaoh Hound | 11-14 | 12.5 | 50 | 50 |
| Plott | 11-13 | 12 | 55 | 47.5 |
| Pointer | 11-15 | 13 | 65 | 55 |
| Polish Lowland Sheepdog | 10-14 | 12 | 40 | 40 |
| Pomeranian | 12-16 | 14 | 5 | 5 |
| Poodle (Standard) | 12-15 | 13.5 | 65 | 45 |
| Portuguese Water Dog | 10-14 | 12 | 51 | 42.5 |
| Pug | 12-15 | 13.5 | 16 | 16 |
| Puli | 10-15 | 12.5 | 30 | 30 |
| Rat Terrier | 16 | 16 | 17.5 | 17.5 |
| Redbone Coonhound | 11 | 11 | 57.5 | 57.5 |
| Rhodesian Ridgeback | 10-12 | 11 | 85 | 70 |
| Rottweiler | 8-11 | 9.5 | 115 | 90 |
| Saluki | 12-14 | 13 | 52.5 | 52.5 |
| Samoyed | 10-12 | 11 | 55 | 42.5 |
| Schipperke | 13-15 | 14 | 13 | 13 |
| Soft-coated Wheaten Terrier | 12-14 | 13 | 37.5 | 32.5 |
| Spinone Italiano | 12-14 | 13 | 75.5 | 64 |
| Staffordshire Bull Terrier | 12-14 | 13 | 33 | 28 |
| Standard Schnauzer | 12-14 | 13 | 42.5 | 42.5 |
| Sussex Spaniel | 11-13 | 12 | 40 | 40 |
| Swedish Vallhund | 13 | 13 | 27.5 | 27.5 |
| Tibetan Mastiff | 11-14 | 12.5 | 120 | 95 |
| Tibetan Spaniel | 14 | 14 | 12 | 12 |
| Tibetan Terrier | 12-15 | 13.5 | 24 | 24 |
| Toy Fox Terrier | 13-14 | 13.5 | 6.5 | 6.5 |
| Treeing Walker Coonhound | 12-13 | 12.5 | 60 | 60 |
| Vizsla | 10-14 | 12 | 57.5 | 49.5 |
| Weimaraner | 10-13 | 11.5 | 80 | 65 |
| Welsh Springer Spaniel | 12-15 | 13.5 | 47.5 | 42.5 |
| Welsh Terrier | 12-14 | 13 | 21 | Prop. smaller |
| West Highland White Terrier | 12-14 | 13 | 17.5 | 17.5 |
| Whippet | 12-15 | 13.5 | 32.5 | 32.5 |
| Wirehaired Pointing Griffon | 12-14 | 13 | 60 | 42.5 |
| Xoloitzcuintle | 13 | 13 | 42.5 | 42.5 |
| Yorkshire Terrier | 14-16 | 15 | 7 | 7 |
| R=-0.71; R^2^ = 0.51 | | | | |

Supplementary Table 3. Relative lifespan and body weight in domestic cats.

| Domestic Cat Breed | Average Lifespan (Years) | Average Weight  (Pounds) |
| --- | --- | --- |
| Abyssinian | 12 | 10 |
| American Bobtail | 14 | 12.5 |
| American Curl | 15 | 7.5 |
| American Shorthair | 17.5 | 12.5 |
| American Wirehair | 9.5 | 11.5 |
| Balinese | 20 | 11.5 |
| Bengal | 14 | 11.5 |
| Birman | 14 | 11 |
| Bombay | 17.5 | 8.5 |
| British Shorthair | 12 | 12 |
| Burmese | 17 | 10 |
| Cornish Rex | 13 | 7.5 |
| Devon Rex | 12 | 7.5 |
| Egyptian Mau | 14.5 | 10 |
| European Burmese | 18.5 | 8 |
| Exotic Shorthair | 13 | 11 |
| Havana Brown | 13.5 | 9 |
| Himalayan | 15 | 9.5 |
| Japanese Bobtail | 16.5 | 9 |
| Javanese | 12.5 | 7.5 |
| Korat | 15 | 8 |
| LaPerm | 12.5 | 8 |
| Maine Coon | 13.5 | 20 |
| Manx | 11 | 10 |
| Norwegian Forest Cat | 15 | 16.5 |
| Ocicat | 12.5 | 10 |
| Oriental | 12.5 | 7.5 |
| Persian | 15 | 9.5 |
| Pixie-bob | 12 | 11 |
| Ragdoll | 14.5 | 14 |
| Russian Blue | 17.5 | 11.5 |
| Scottish Fold | 15 | 11 |
| Selkirk Rex | 12.5 | 12.5 |
| Siamese | 17.5 | 9 |
| Siberian or | 13 | 15 |
| Singapura | 12 | 6 |
| Somali | 11 | 10.5 |
| Sphynx | 14 | 11 |
| Tonkinese | 13 | 9 |
| Turkish Angora | 15 | 7.5 |
| Turkish Van | 14.5 | 12.5 |
| R=0.006820545 | | |
